# Incidence and Outcomes of Patients with Functionally Univentricular Heart Born in Latvia, 2007 to 2015

**DOI:** 10.3390/medicina54030044

**Published:** 2018-06-11

**Authors:** Katrina Rutka, Inguna Lubaua, Elina Ligere, Amanda Smildzere, Valts Ozolins, Reinis Balmaks

**Affiliations:** 1 Clinic of Anesthesiology and Reanimatology, Pauls Stradins Clinical University Hospital, Riga, Latvia, Pilsonu Street 13, LV-1002 Riga, Latvia; katrina.rutka@rsu.lv; 2 Department of Anesthesiology and Reanimatology, Riga Stradins University, Riga, Latvia, Pilsonu Street 13, LV-1002 Riga, Latvia; 3 Department of Pediatrics, Riga Stradins University, Latvia, Vienibas Gatve 45, LV-1004 Riga, Latvia; inguna.lubaua@rsu.lv (I.L.); elina.ligere@rsu.lv (E.L.); 4 Clinic for Pediatric Cardiology and Cardiac Surgery, Children’s Clinical University Hospital, Riga, Latvia, Vienibas Gatve 45, LV-1004 Riga, Latvia; valts.ozolins@bkus.lv; 5 Neonatal Intensive Care Unit, Children’s Clinical University Hospital, Riga, Latvia, Vienibas Gatve 45, LV-1004 Riga, Latvia; amanda.smildzere@bkus.lv; 6 Clinical Skills and Medical Technology Department, Riga Stradins University, Latvia, Anninmuizas bulvaris 26a, LV-1067 Riga, Latvia; 7 Intensive Care Unit, Children’s Clinical University Hospital, Riga, Latvia, Vienibas Gatve 45, LV-1004 Riga, Latvia

**Keywords:** functionally univentricular heart, hypoplastic left heart syndrome, outcomes

## Abstract

*Background and Objectives*: A functionally univentricular heart is the term used to describe congenital heart defects where it is impossible to restore two pumping chambers. These lesions are associated with high mortality, morbidity, and medical resource utilization. The aim of this study was to review incidence and outcomes of patients with a functionally univentricular heart at the only pediatric cardiac surgery center in Latvia. *Methods*: We performed a retrospective review of medical records of (i) all children with a functionally univentricular heart treated at the Clinic of Pediatric Cardiology and Cardiac Surgery, and (ii) all prenatally diagnosed cases of univentricular heart at Children’s Clinical University Hospital in Latvia. We reviewed data regarding children born from January 1, 2007, to December 31, 2015. The children’s cardiac anatomy and interventions were categorized in accordance with the International Pediatric and Congenital Cardiac Code (v3.3). *Results*: During the study period, 49 patients with a functionally univentricular heart were admitted to Children’s Clinical University Hospital with a corrected incidence of 0.69 per 1000 live births per year. There were 26 patients that had a hypoplastic left ventricle, and 22 patients that had a hypoplastic right ventricle, while one patient had an indeterminate ventricle. Thirty (61.2%) patients had died by the end of data collection. Twenty-one of the 30 deaths occurred before or immediately after stage I surgical palliation. Cumulative neonatal and 5-year survival of patients with a hypoplastic right ventricle was 81.8% and 63.6%, respectively; for patients with hypoplastic left ventricle—46.2% and 17.3%, respectively. *Discussion*: This is the first mid-term outcome study of patients with a univentricular heart in Latvia. The high mortality reflects the challenges of a small-volume, developing congenital cardiac surgery center. Data from this study will be used as a baseline for quality improvement.

## 1. Introduction

Congenital heart disease (CHD) is the most frequent congenital disorder in newborns, occurring with a frequency of approximately 1 in 40 neonates [1]. Some CHDs are non-critical; however, about 25% are critical and will require immediate intervention following birth [2]. Amongst these, there is a group of diagnoses called ‘functionally univentricular heart’, which are congenital cardiovascular malformations where it is impossible to restore normal physiology with one ventricular pump committed to the systemic and another to the pulmonary circulation [3]. Functionally univentricular heart has one of the highest mortality rates among congenital birth defects. These children require many resources and present many management challenges to a multidisciplinary clinical team. Examples of functionally univentricular heart include hypoplastic left heart syndrome (HLHS), double-inlet left ventricle, tricuspid atresia, and other less frequent cardiac malformations where biventricular surgical repair is impossible [3,4].

Estimates of long-term morbidity and survival are required to adequately assess the variety of health and social services required for patients with a functionally univentricular heart during their life-time. Several population-based studies have been published from Western Europe and North America [5], however it has been shown that outcomes in an Eastern European center can be largely different from the results in resource-rich countries [6]. No long-term outcome studies regarding patients with a univentricular heart have been conducted in Latvia. The purpose of this study was to review the incidence and outcomes of patients with a functionally univentricular heart treated at the only pediatric cardiac surgery center in Latvia.

## 2. Materials and Methods

This study was approved by the Ethics Committee of the Children’s Clinical University Hospital (Decision No. 5-2016; 12 September 2016); given the retrospective nature and absence of any patient identification, the requirement for individual patient or parent consent was waived.

In Latvia, all children with CHD receive state-funded public healthcare, and all of these patients are treated exclusively at Children’s Clinical University Hospital in Riga.

The registry held by Clinic of Pediatric Cardiology and Cardiac Surgery at the Children’s Clinical University Hospital contains data of all children diagnosed with congenital heart disease, including the majority of antenatally diagnosed cases. We searched this registry for all the patients born in Latvia from 1 January 2007, until 31 December 2015, with a functionally univentricular heart according to the definition from the International Society for Nomenclature of Pediatric and Congenital Heart Disease. Information on date of birth, type of CHD, existence and timing of prenatal diagnosis, date and timing of palliative surgical repair operations, survival status, and time of death, if applicable, was obtained from electronic medical records of patients at the Children’s Clinical University Hospital. Cardiac anatomy and interventions were categorized in accordance with the International Pediatric and Congenital Cardiac Code (v3.3). Each case was reviewed by two experienced pediatric cardiologists to ensure correct classification and data completeness.

Incidence calculations were based on publicly available birth rate data assembled and published by the Centre for Disease Prevention and Control of Latvia; a total of 193,223 children were born in Latvia during the study period.

Descriptive statistics and basic calculations were done by using Microsoft Excel software (v15.26). Cumulative survival rates were obtained by using the Kaplan-Meier method in SPSS Statistics (v24.0) software. Log-rank (Mantel-Cox) test was used to compare survival distributions of two groups; statistical significance was defined as *p* < 0.05.

## 3. Results

During the study period, 111 cases of functionally univentricular heart were diagnosed prenatally, and pregnancies were terminated in 85 (76.6%) of these cases. In the same time period, 49 newborns with functionally univentricular heart were admitted to the Children’s Clinical University Hospital; 25 (51.1%) of these patients had prenatal confirmation or suspicion of a CHD.

There were 26 patients that had a hypoplastic left ventricle, 22 patients had a hypoplastic right ventricle, and one patient had an indeterminate ventricle. Table 1 shows the number of patients in each of the anatomical categories and information on their cardiac morphology. There were 19 patients with hypoplastic left heart syndrome, which represented the largest group.

**Figure 1:**
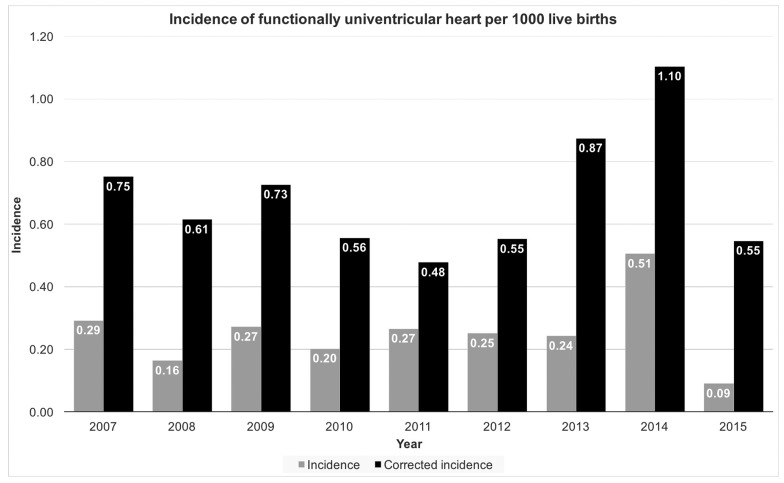
Incidence of functionally univentricular heart per 1000 live births. Grey columns indicate total incidence; black columns indicate corrected incidence, after adjusting for terminated pregnancies.

The average incidence of functionally univentricular heart was 0.25 cases per 1000 live births per year; after adjusting for terminated pregnancies, the corrected average incidence was 0.69 per 1000 live births per year (see Figure 1 for annual incidence rates).

During the study period, 39 patients underwent a total of 56 palliative surgical interventions. These included 34 stage I, 18 stage II, and 4 stage III procedures (Table 2). The most frequently performed stage I procedure (12 patients) was the hybrid approach consisting of stent placement in the arterial duct, with or without banding of pulmonary artery branches. The majority of stage II procedures performed at Children’s Clinical University Hospital (14 patients) were bidirectional cavopulmonary (Glenn) anastomoses. Four patients underwent total cavopulmonary connection (Fontan procedure) as a stage III palliation.

**Figure 2:**
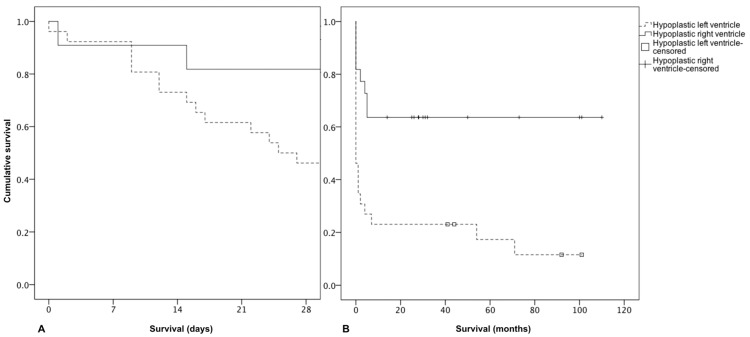
Kaplan-Meier estimate of neonatal (**A**) and overall cumulative (**B**) survival for the patients with functionally univentricular hearts according to the hypoplastic ventricle. Solid lines represent patients with a hypoplastic right ventricle; interrupted lines represent patients with a hypoplastic left ventricle.

There were 30 (61.2%) patients that had died by the end of data collection. The majority of deaths (21 out of 30) occurred before stage I surgical palliation (8 patients), or intraoperatively (7 patients), or in the immediate post-operative period (6 patients). The cumulative neonatal survival of patients with a hypoplastic right ventricle or hypoplastic left ventricle was 81.8% and 46.2%, respectively (*p* = 0.003; Figure 2), while the cumulative five-year survival was 63.6% and 17.3%, respectively (*p* = 0.002; Figure 2). The difference in cumulative survival rates calculated for patients with and without prenatally diagnosed CHD did not reach statistical significance (data not shown). The number of patients alive at one year of age for the most common subtypes of morphology were as follows: HLHS: 4 out of 19, right isomerism: 1 out of 4, pulmonary atresia with intact ventricular septum: 3 out of 5, tricuspid atresia: 5 out of 8, and double-inlet left ventricle: 6 out of 7.

**Figure 3:**
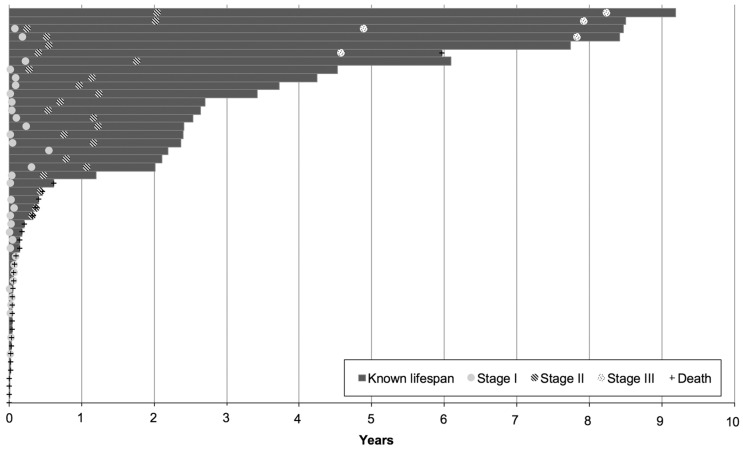
The timing of surgical interventions and time of death. Bars represent the known lifespan of individual patients; crosses indicate death; filled, striped, and dotted circles represent stage I, II, and III interventions, respectively.

The timing of surgical interventions and time of death is represented in Figure 3.

## 4. Discussion

In this retrospective study, we measured epidemiology and outcomes, and audited the current practice of treatment for functionally univentricular congenital heart disease. Children’s Clinical University Hospital is the single tertiary children’s hospital and home of the only pediatric cardiology and cardiac surgery, as well as tertiary pediatric and neonatal intensive care services in the country. By definition, it is a small volume cardiac center, and the number of cardiopulmonary bypass surgeries averages 50 cases per year [7,8,9]. Only since 2008 have surgeries been offered to patients with complex pathology, such as a functionally univentricular heart.

The current pathway of care for a functionally univentricular heart in Latvia is as follows: (i) children with an antenatal diagnosis are delivered in a specialized maternity ward, and immediately after, are transferred to Children’s Clinical University Hospital; (ii) for the majority of patients with a ductal-dependent functionally univentricular heart, a hybrid approach is used where the arterial duct is stented (with or without limiting pulmonary blood flow) until the child gains more weight for the definitive surgery, or in most complicated cases, until the child is transferred abroad to more experienced cardiac surgery centers. Typically, subsequent stage II and III palliations and follow-up are done in Latvia; however, some patients continue to receive full care abroad.

Similar to that reported in the Congenital Heart Surgery Database of the European Association for Cardio-Thoracic Surgery (EACTS) and the Society of Thoracic Surgeons (STS), three of the most common diagnoses we observed in patients with a functionally univentricular heart were hypoplastic left heart syndrome, tricuspid atresia, and double-inlet left ventricle [3].

Prenatal diagnosis and termination rates have a significant effect on the incidence of critical CHDs [10]. Using the same classification, the mean corrected incidence rate of univentricular heart (live births and terminated pregnancies) in the Danish Register of Congenital Heart Disease was 0.39 per 1000 live births per year [11], very similar to the combined unadjusted incidence of most common univentricular heart lesions in the study by Samanek and Voriskova [12]. Comparatively, the corrected incidence in our study was 0.69 per 1000 live births per year. It is likely that this difference is due to the low total numbers and the rarity of the disease [1]. The percentage of terminated pregnancies we observed in our cohort was very high at 76.6%; higher than the 66.9% reported in the UK in 1999 [10], and even more so than the 34.6% reported in Denmark from 2000 until 2009 [11]. A hypothetical explanation for the higher termination rate is that since Latvia joined the European Union, many of its citizens of reproductive age work and give birth abroad, but return home for medical treatment. It is therefore very important to interpret incidence data by adjusting for terminated pregnancies as this practice is very variable.

As in other cohorts [11,13,14], most of the deaths in the current study occurred in the first few months of life, and after the first year of life, the survival curves plateaued, which can be explained by the survival of children with a more favorable anatomy and without extra major cardiac defects. It also indicates the critical importance of meticulous intensive care in the neonatal period and timely interventions.

The higher cumulative survival rate observed in children with a hypoplastic right ventricle complies with the evidence that the left ventricle is better suited to be the single functioning ventricle ensuring blood flow in the systemic circulation. A particular challenge is posed by patients with hypoplastic left heart syndrome, requiring the high-risk Norwood type aortic arch reconstruction with cardiopulmonary bypass and circulatory arrest. Our calculated five-year survival rate for patients with hypoplastic left heart syndrome was only 11.5% (data not shown). This is similar to the 5-year survival rate (12.5%) in a meta-analysis of several population-based studies [5]. Over the last decade, the surgical results in Western Europe and North America have dramatically improved, and the 30-day survival for HLHS is about 90% [15]. It is important to note that all children in Latvia were offered surgical treatment, and the high mortality is not because they had chosen comfort care only. In addition, we do not believe that this high mortality rate could be explained by the hybrid strategy, because some studies have demonstrated a short-term survival benefit when compared to the more traditional Norwood approach [16,17,18]. Although we were not able to calculate the cumulative 5-year survival of other anatomical variants of univentricular heart due to a low number of patients, as expected, patients with right isomerism and pulmonary atresia with intact ventricular septum had high mortality.

Multiple studies have demonstrated an inverse relationship between pediatric cardiac surgery case volume and patient mortality [19], and the mortality appears to be the lowest when the volume of the center reaches at least 200 cases per year [9,20]. Therefore, regionalization of cardiac patients is increasingly important as case complexity increases. Taking into account the relatively small populations of the individual countries located in the Baltic region, it is important to develop cross-border collaboration to improve outcomes of patients with functionally univentricular hearts, as shown by the collaboration between Slovenia and Slovakia with regard to surgical treatment of transposition of the great arteries [21]. In addition to surgical skill and experience, outcomes are highly dependent on meticulous pre- and post-operative intensive care [20,22], and this is probably where the first steps towards quality improvement needs to be taken in Latvia.

The strengths of our study include its population-based nature, review of our detailed database in Latvia, and a follow-up period of up to 9 years. Moreover, identification of children with a functionally univentricular heart did not rely on administrative coding, but resulted from a clinically classified data set, which minimized the potential for misclassification.

This study also had several limitations. Firstly, although the Children’s Clinical University Hospital is the only pediatric cardiac surgery center in Latvia, and this study represents most of the patients in the given time period, there may be some exceptions. For example, fetal echocardiography is also performed at smaller clinics, and termination of these pregnancies, if performed, would not appear in our registry. This is particularly problematic for this study in cases where the reason for termination is a severe extra cardiac defect. Secondly, care for newborns during the first 7 days of life, unless tertiary interventions are required, is primarily done at regional perinatal care centers. Therefore, it is possible that some infants may have died before transfer and have not been accounted for. Thirdly, we did not assess the effect of extra cardiac defects on mortality. Fourthly, we did not have data on other long-term outcomes, such as heart failure later in life. Finally, the sample size of this study was small and caution must be taken when making prognosis of an individual patient as it will largely depend on the specific morphology.

## 5. Conclusions

This is the first long-term outcome study of patients with a functionally univentricular heart in Latvia. The high mortality reflected the challenging nature of these heart defects, particularly at a small-volume congenital cardiac surgery center during a learning curve period. Taking into account the average incidence of this CHD and the national birth-rate, it can be expected that five to six new patients with this type of CHD will require treatment in Latvia each year, thus, limiting the ability to obtain experience in the management of these patients locally. This retrospective study provides data that may be used to estimate the prognosis of these patients in Latvia, allowing clinicians and parents to make informed decisions during their long and complex treatment. This information could also be used to plan national treatment strategies and justify the transfer of these patients to larger cardiothoracic centers abroad.

## Figures and Tables

**Table 1:** Number of patients according to cardiac anatomy.

International Pediatric and Congenital Cardiac Code (v3.3)	Diagnosis	Dominant Ventricle	No of Patients
HLHS	HLHS	Right	19
Single ventricle, Tricuspid atresia	TA, VSD	Left	6
	TA, VSD, TGA	Left	2
Single ventricle, Other	PA-IVS	Left	4
	PS-IVS, Ebstein’s anomaly	Left	1
	DORV, PA, VSD	Right	1
	Ebstein’s anomaly, PS	Left	1
	Double-outlet indeterminate ventricle, VSD, TGA	Indeterminate	1
Single ventricle, DILV	DILV, VSD, TGA	Left	3
	DILV	Left	2
	DILV, DORV	Right	1
	DILV, VSD, TGA, DORV	Left	1
Single ventricle, Heterotaxy syndrome	Heterotaxy syndrome, right isomerism, TAPVC, Unbalanced AV canal, DORV, PS	Right	1
	Heterotaxy syndrome, right isomerism, TAPVC, Unbalanced AV canal, DORV, TGA, PS	Right	1
	Heterotaxy syndrome, right isomerism, TA, TAPVC	Left	1
Single ventricle, Mitral atresia	MA, VSD, DORV	Right	1
	MA, VSD, Truncus arteriosus	Right	1
Single ventricle + TAPVC	TA, VSD, TAPVC, right isomerism	Left	1
Single ventricle, Unbalanced AV canal	TOF, Unbalanced AV canal	Right	1

ASD: atrial septal defect; DILV: double-inlet left ventricle; DORV: double-outlet right ventricle; HLHS: hypoplastic left heart syndrome; MA: mitral atresia; PA: pulmonary atresia; PA-IVS: pulmonary atresia with intact ventricular septum; PS: pulmonary stenosis; TA: tricuspid atresia; TAPVC: total anomalous pulmonary venous connection; TGA: transposition of the great arteries; TOF: tetralogy of Fallot; VSD: ventricular septal defect.

**Table 2:** List and number of surgical palliation interventions.

Stage of Palliation	Intervention	No of Procedures ^1^
Stage I	Hybrid approach “stage I”: stent placement in arterial duct + application of right and left pulmonary artery bands	12
	Pulmonary artery banding	8
	Hybrid approach “stage I”: stent placement in arterial duct	4
	Modified Blalock-Taussig shunt	4
	Central aortopulmonary shunt	3
	Norwood procedure	2
	HLHS biventricular repair	1
Stage II	Bidirectional cavopulmonary anastomosis (Glenn)	14
	Hybrid approach “stage II”: Norwood procedure + superior cavopulmonary anastomosis + pulmonary artery debanding	2
	hemi-Fontan	1
	Superior cavopulmonary anastomosis (Glenn or hemi-Fontan) + atrioventricular valvuloplasty	1
Stage III	Fontan, total cavopulmonary connection, external conduit, fenestrated	3
	Fontan, total cavopulmonary connection, lateral tunnel, fenestrated	1

^1^ Four patients in the study were referred to other centers for some or all of their surgeries; these procedures are not included here.
